# Recombinant Erythropoietin in Humans Has a Prolonged Effect on Circulating Erythropoietin Isoform Distribution

**DOI:** 10.1371/journal.pone.0110903

**Published:** 2014-10-21

**Authors:** Niels Jacob Aachmann-Andersen, Søren Just Christensen, Kristian Lisbjerg, Peter Oturai, Anne-Kristine Meinild-Lundby, Niels-Henrik Holstein-Rathlou, Carsten Lundby, Niels Vidiendal Olsen

**Affiliations:** 1 Department of Neuroscience and Pharmacology, University of Copenhagen, Copenhagen, Denmark; 2 Clinic of Clinical Physiology, Nuclear Medicine and PET, Centre of Clinical Investigation, Rigshospitalet, Copenhagen, Denmark; 3 Center for Integrative Human Physiology (ZIHP), University of Zurich, Institute of Physiology, Zürich, Switzerland; 4 Department of Biomedical Sciences, University of Copenhagen, Copenhagen, Denmark; 5 Department of Neuroanaesthesia, The Neuroscience Centre, Rigshospitalet, Copenhagen, Denmark; University of Leuven, Belgium

## Abstract

The membrane-assisted isoform immunoassay (MAIIA) quantitates erythropoietin (EPO) isoforms as percentages of migrated isoforms (PMI). We evaluated the effect of recombinant human EPO (rhEPO) on the distribution of EPO isoforms in plasma in a randomized, placebo-controlled, double-blinded, cross-over study. 16 healthy subjects received either low-dose Epoetin beta (5000 IU on days 1, 3, 5, 7, 9, 11 and 13); high-dose Epoetin beta (30.000 IU on days 1, 2 and 3 and placebo on days 5, 7, 9, 11 and 13); or placebo on all days. PMI on days 4, 11 and 25 was determined by interaction of N-acetyl glucosamine with the glycosylation dependent desorption of EPO isoforms. At day 25, plasma-EPO in both rhEPO groups had returned to values not different from the placebo group. PMI with placebo, reflecting the endogenous EPO isoforms, averaged 82.5 (10.3) % (mean (SD)). High-dose Epoetin beta decreased PMI on days 4 and 11 to 31.0 (4.2)% (p<0.00001) and 45.2 (7.3)% (p<0.00001). Low-dose Epoetin beta decreased PMI on days 4 and 11 to 46.0 (12.8)% (p<0.00001) and 46.1 (10.4)% (p<0.00001). In both rhEPO groups, PMI on day 25 was still decreased (high-dose Epoetin beta: 72.9 (19.4)% (p = 0.029); low-dose Epoetin beta: 73.1 (17.8)% (p = 0.039)). In conclusion, Epoetin beta leaves a footprint in the plasma-EPO isoform pattern. MAIIA can detect changes in EPO isoform distribution up til at least three weeks after administration of Epoetin beta even though the total EPO concentration has returned to normal.

## Introduction

The use of recombinant human erythropoietin (rhEPO) for enhancement of sports performance was banned in 1990 by the International Olympic Committee. Still, however, doping by blood manipulation remains a problem in elite athletic competitions in spite of ever-increasing anti-doping efforts [1]–[4]. The performance gain following the increase in hemoglobin mass is maintained for at least three weeks [1], [2]. This in combination with short elimination half-lifes of most rhEPO isoforms makes it possible to accommodate doping practices in relation to competition events so as to avoid direct detection of a former use of rhEPO [1], [5]. Moreover, rhEPO isoforms differ only slightly from the endogenous isoforms by their posttranslational glycosylation pattern [3], [6]. Several tests have been developed to distinguish between specific forms of EPO glycosylation but valid and sensitive tests are lacking [1], [3], [7]. Because of the shortcomings of direct tests, indirect marker methods, referred to as ‘Blood Passport’ or the World Anti-Doping Association's (WADA) ‘Athlete Biological Passport’, have been introduced [5]. These methods are based on personalized monitoring of haematological parameters, but testing in controlled trials has showed a high incidence of false-negative results [8]–[10].

A new promising approach to anti-doping testing has been the introduction of the membrane-assisted isoform immunoassay (MAIIA) which is a combination of lectin affinity chromatography and lateral flow immunoassay in a combined test-strip [11]. For identification of EPO isoforms, the method uses a non-selective lectin (wheat germ agglutinin (WGA)). In the next step, the addition of buffers containing a competing monosaccharide (N-acetylglycosamine (GlcNAc)) allows a separation of EPO isoforms in fractions according to the specific glycosylation: at high concentrations of GlcNAc (>100 mM), all EPO isoforms are eluted from the lectin. At lower concentrations of GlcNAc (<20 mM), however, only fractions are released from the WGA zone, the amount depending on their glycosylation. Eluted EPO is then bound to an immobilized anti-EPO antibody and quantified by the use of a second anti-EPO antibody labelled with carbon black nano-strings. By testing the same plasma sample at both a high GlcNAc concentration (total desorption of all isoforms) and a low GlcNAc concentration (fractional specific desorptions of given isoforms), the method allows calculation of the ‘percent of migrated isoforms’ (PMI) [12]. PMI is the amount of EPO desorbed at a low GlcNAc concentration as a percentage of the amount desorped at the high GlcNAc concentration [7], [12].

In example, with the use of 15 mM and 300 mM GlcNAc, PMI in a normal plasma sample with only endogenous EPO isoforms average 70–80% [12]. In contrast, PMI in a solution of Epoetin beta, a form of rhEPO synthesized in Chinese hamster ovary cells, is around 30 % [12]. In samples from subjects that were injected with Epoetin beta (5,000 IU) every second day for 14 days and then again at days 21 and 28, PMI decreased to a nadir of around 30 % at day 16 [13]. Seven days after the last injection, when the plasma concentration of EPO had returned to normal, PMI was still decreased compared with baseline values [13]. Interestingly, studies suggest that the resolution between PMIs for EPO subpopulations can be optimized by the use of different low concentrations of GlcNAc. PMI from normal plasma is best distinguished from samples with Epoetin beta at GlcNAc concentrations of 15 mM, whereas lower concentrations may be used to distinguish endogenous EPO isoforms [12].

Thus, MAIIA may provide a sensitive measure of changes in the EPO isoform distribution pattern caused by former use of recombinant EPO even after normalization of total EPO concentrations in plasma. Such a change may be secondary to prolonged suppression by rhEPO of the endogenous renal synthesis of EPO [14], [15] and also remaining small amounts of rhEPO may alter the distribution profile of circulating EPO. In this placebo-controlled, double-blinded, cross-over study, we tested whether MAIIA may be used as an indirect marker method to detect former administration of Epoetin beta.

## Methods and Materials

### Subjects

Sixteen healthy male volunteers (age 25.4 (3.6) years, (mean (SD)), height 183.5 (6.6) cm, body weight 76.9 (7.3) kg and body mass index 22.9 (2.7) kg/m^2^) were included in the study. Before inclusion each subject underwent a medical examination and had to fulfill the following inclusion criterias: male gender, age between 20 and 40 years, non-smoking, arterial systolic blood pressure below 140 mmHg and diastolic blood pressure below 90 mmHg, no actual medication, and body mass index <25. Exclusion criterias were participation in other studies, history of elite athletic performance, history of neoplastic diseases, polycythemia, epilepsy or allergy to rhEPO, and/or exposure to altitude (>1,500 metres above sea level) within three months prior to the study.

### Experimental Protocol

The effect of both low and high doses of rhEPO (Epoetin beta, NeoRecormon, Roche, Welwyn Garden City, UK) and placebo was evaluated in each subject by a randomized, double-blinded, placebo-controlled, cross-over design with a 5 week washout interval between the series:

1. Low dose rhEPO: 5,000 IU (∼65 IU.kg^−1^) of subcutaneously administered Epoetin beta every second day for two weeks (days 1, 3, 5, 7, 9, 11 and 13; placebo on day 2).
2. High dose rhEPO: 30,000 IU (∼390 IU.kg^−1^) of subcutaneously administered Epoetin beta every day for three days (days 1, 2 and 3; placebo on days 5, 7, 9, 11 and 13).
3. Placebo (sodium chloride, isotonic 9 mg/ml, B.Braun, Melsungen, Germany) administered subcutaneously on days 1, 2, 3, 5, 7, 9, 11 and 13.

The primary endpoint in all series was changes in percent migrated isoforms (PMI) at days 4, 11 and 25 as a measure of EPO isoform distribution.

Randomization for the entire study (all three test series) was done before the beginning of the experiment. An independent investigator (NVO) generated a restricted randomization list and after inclusion each subject where given a number and a code, unique for subject and intervention. It was not possible for the subjects to visually distinguish between placebo or study medication. Randomization lists were sealed and not available for persons involved in endpoint registration. NVO did not participate in subject inclusion, study management, end-point evaluation or data analysis. The study was approved by the Regional Committee on Biomedical Research Ethics, Committee B (protocol no. H-2-2011-068) and the Danish Medicine Agency (EudraCT 2001-005137-39). The study was conducted according to the principles of Good Clinical Research (GCP), monitored by the GCP Unit at Copenhagen University Hospital (Bispebjerg Hospital) and registered on www.clinicaltrials.gov (NCT01584921). All subjects were informed verbally and in writing before the beginning of the study and gave written informed consent to participate. All experiments were done at the Copenhagen University Hospital (Rigshospitalet), Denmark.

All injections were administered subcutaneously between 08.00 and 11.00 a.m. The subjects did not receive any iron supplementation as normally administered during treatment for anemia or in doping so as to avoid confounding effects regarding the effect of Epoetin beta *per se*. Three days before each test day subjects refrained from excessive salt intake, according to written instructions from a dietitian. The subjects refrained from alcohol, caffeine-containing drinks and extensive exercise 24 hours before each test day. These measures were due to concomitant metabolic and renal clearance studies reported elsewhere. Plasma samples were collected on day 4, 11 and 25. Blood samples were obtained from an anticubital vein after at least 180 min of rest in a sitting position using EDTA tubes and before administration of rhEPO. Immediately after centrifugation at 3000 g for 10 minutes, the plasma were stored at −80°C until analysis.

### Total EPO concentration

Plasma EPO concentrations were measured by means of the commercially available Quantikine IVD ELISA kit (R&D Systems Europe, Ltd., Abingdon, UK) according to the manufacturer's protocol which is based on the double-antibody sandwich method. Assay results were measured spectrophotometrically at 450 nm using a microplate reader to determine the optical density. Duplicate readings were averaged for each standard, control and specimen. The log of erythropoietin concentration was plotted *versus* the log of optical density for the standard curve. Concentrations are given in mIU/ml; samples with EPO concentrations above the range of the assay (2.5–200 mIU/mL) were diluted ten-fold before a second analysis.

### EPO isoform distribution

The change in EPO isoform distribution was measured by membrane-assisted isoform immunoasssay (MAIIA) [13]. The MAIIA kit is based on affinity chromatography and lateral flowimmunoassay on a strip with two important zones: a wheat germ agglutinin (WGA) zone that interacts with EPO via its sugar moieties and, downstream from this, an anti-EPO zone that immobilizes EPO. The strip was immersed in a well containing the sample, which migrates in the strip due to capillary forces. N-acetyl glucosamine (GlcNAc) in different concentrations were used to release the different EPO isoforms from the WGA interaction. With high GlcNAc concentration (300 mM) all EPO melocules are released from the WGA zone but with lower concentrations (5, 10, 15 or 20 mM) different isoforms of EPO are released depending on their glycosylation pattern [11]–[13]. Calculation of the percentage of migrated isoform (PMI) was done by analysing each sample with several strips using both high and low GlcNAc concentrations. For detection and quantification of EPO the anti-EPO zone was used in combination with a secondary anti-EPO antibody and carbon black nano-strings.

**Figure 1:**
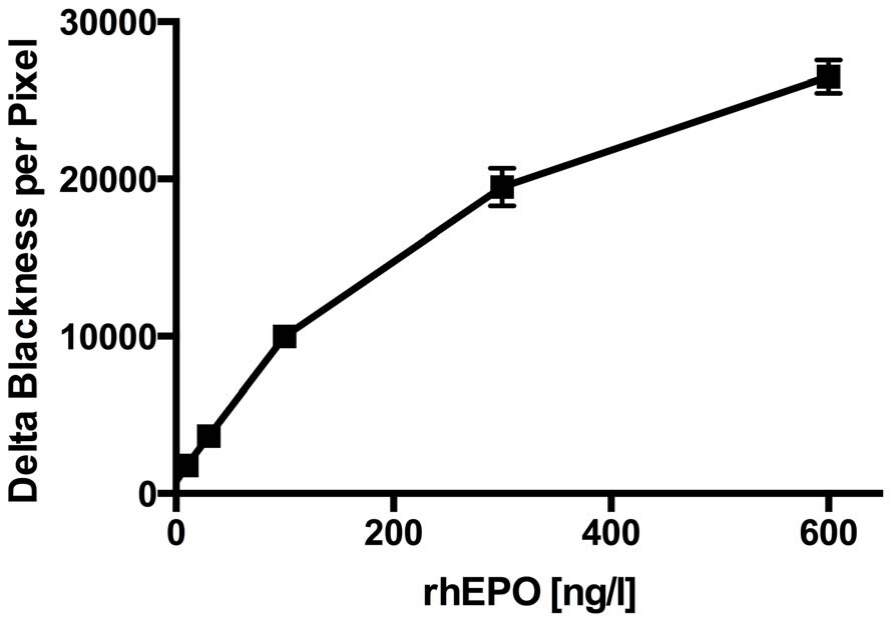
Standard curve. Concentrations of rhEPO (Epoietin beta) were 0, 10, 30, 100, 300 and 600 ng/l. All solutions were tested at 300 mM of GlcNAc. Values are means ± SD. N = 6.

**Figure 2:**
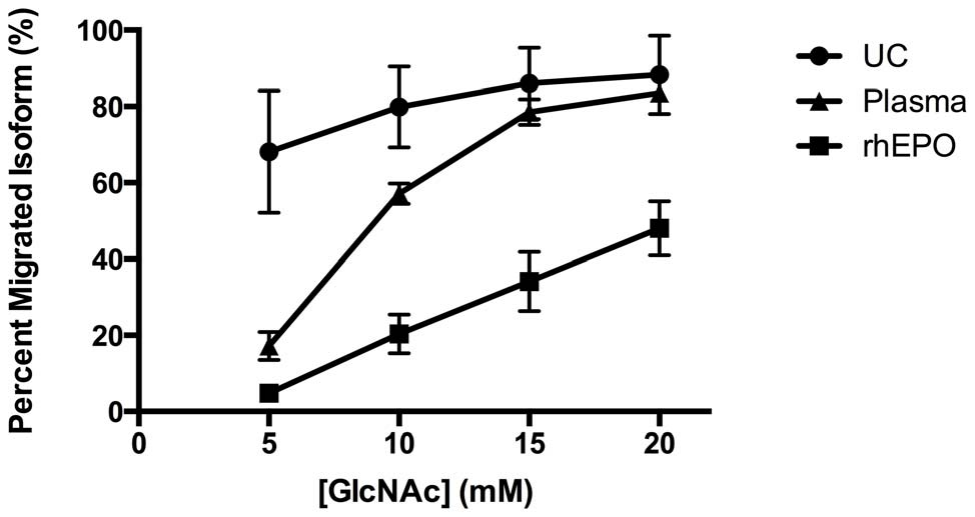
Optimization curve. For optimization of W elution buffer *low*, three samples were used: 1) UC, umbilical cord sample; 2) Plasma sample form a normal subject; and 3) rhEPO, epoetin beta (300 ng/l). All samples from the three specimen were tested with GlcNAc concentrations of 5 mM, 10 mM, 15 mM, 20 mM, and 300 mM. Values are means (SD). N = 6 for UC, N = 5 for plasma and N = 5 for rhEPO.

The MAIIA kit (EPO purification kit, MAIIA Diagnostics, Uppsala, Sweden) was used to purify EPO plasma samples. The procedure was done according to the directions of the manufactor; 1.0 ml of plasma was purified on single use anti-Epo columns and eluted into a final volume of 55 µl and stored at −20°C until analysis. A standard curve was made and the optimal concentration of W elution buffer *low* was determined. The standards had concentrations of Epoetin beta of 0, 10, 30, 100, 300 and 600 ng/l and were all analysed at 300 mM (high) GlcNAc. The final standard curve (Figure 1) represents a mean of four standard curves and the standard curve parameters were obtained by the use of a four parameter logistic curve fitting routine to fit the “Delta Blackness per Pixel” (DBpB). The parameters were entered in an excel template provided by MAIIA and used for calculation of PMI. For optimization of W elution buffer *low* (Figure 2), three sets of samples were used: 1) a solution of Epoetin beta (300 ng/l); 2) a plasma sample from a normal subject (CL); and 3) arterial plasma samples obtained from umbillical cord (N = 6), as described previously [16]. All sets of samples from the three specimen were tested with GlcNAc concentrations of 5 mM, 10 mM, 15 mM, 20 mM, and 300 mM of GlcNAc. Samples were diluted 50, 20, 10 or 5 times in a dilution buffer, aming for a sample concentration of EPO within the range of the assay (10–500 ng/l). Based on the results from the optimization analyses (Figure 2), all plasma samples from days 4, 11 and 25 were analysed in duplicates and under standardized conditions regarding temperature, humidity, scanner and reagent lot number at 15 mM and high GlcNAc. In addition, plasma samples from days 11 and 25 were analysed in duplicate at 5 mM and high GlcNAc.

### Statistical analysis

Statistical analyses were done by the use of the statistical SPSS software (IBM SPSS Statistics, version 20.0.0). Entries into the database of all data were verified by the GCP Unit at Copenhagen University Hospital. To assess the effect of rhEPO treatment on total EPO concentration and PMI values a linear mixed-effects model (MIXED) for repeated measurements was used. (SPSS: Analyze>Mixed Model>Linear). Repeated measures and pairwise comparisons *versus* baseline were corrected for multiple comparisons (Bonferroni). Results are expressed as means (SD). All calculations regarding plasma EPO concentrations and PMI values on day 4, 11 and 25 were done comparing each treatment and day against the mean of placebo values on day 4, 11 and 25. No significant carry-over or period effect was found for any of the endpoints. The assumption of normal distribution was evaluated by visual inspection. If necessary, data was log_10_ transformed before analysis. Two-sided significance tests were used with a significance level of 5 %.

## Results

All subjects received 100 % of the planned injections. One subject was excluded due to illness commencing during the first 5-week wash-out period between the first and second series. It was not characterized as an adverse effect by the GCP unit, and the subject's data from the first series are included in the statistical calculations.

### Total plasma EPO concentration (Table 1)

**Table 1:** Total plasma EPO concentration (mIU/ml).

	Day 4	Day 11	Day 25
Placebo	8.1 (2.6)	11.3 (3.9)	10.1 (3.0)
Low-dose EPO	29.7 (11.2)**	25.5 (8.6)**	8.1 (3.8)
High-dose EPO	470.3 (202.0)**	15.4 (6.2)*	8.3 (4.9)

N = 15. Values are means (SD). Data was log_10_ transformed before analysis.

*p = 0.004;

**p<0.00001 compared with placebo.

Total plasma EPO concentration increased after administration of both low-dose and high-dose Epoetin beta. Compared with placebo, low-dose Epoetin beta increased the total plasma EPO concentration by 3-fold on days 4 (p<0.00001) and 11 (p<0.00001) whereas values on day 25 did not differ from placebo (p = 0.116). Concentrations after low-dose Epoetin beta did not differ between days 4 and 11 (p = 0.65) but on both days values were higher compared with day 25 (p<0.00001). High-dose Epoetin beta increased the total plasma EPO concentration by 48-fold (p<0.00001) and two-fold (p = 0.004) at days 4 and 11 whereas values on day 25 did not differ from placebo (p = 0.121). There was significant difference between days 4, 11 and 25 within the high-dose intervention (p<0.00001).

### Haematological variables (Table 2)

**Table 2:** Haematological parameters after 4, 11 and 25 days of either placebo, low-dose rhEPO, or high-dose rhEPO.

	Day 4	Day 11	Day 25
Hematocrit (%)			
Placebo	42.9 (1.9)	40.7 (2.5)	41.4 (2.7)
Low-dose EPO	42.8 (2.0)	43.0 (2.1)	43.5 (2.6)*
High-dose EPO	43.0 (1.5)	43.3 (2.4)*	42.6 (2.8)
Hemoglobin (g⋅dl^−1^)			
Placebo	8.9 (0.4)	8.6 (0.5)	8.6 (0.5)
Low-dose EPO	9.0 (0.4)	8.9 (0.4)	8.9 (0.5)
High-dose EPO	9.0 (0.4)	9.0 (0.5)	8.8 (0.6)
Reticulocytes (×10^9^⋅l^−1^)			
Placebo	31.1 (10.2)	37.9 (11.8)	35.5 (12.4)
Low-dose EPO	40.5 (6.1)	78.7 (19.9)**	26.3 (6.5)
High-dose EPO	68.4 (15.6)**	84.4 (18.0)**	27.9 (8.8)
Ferritin (µg⋅l^−1^)§			
Placebo	85.2 (64.1)	70.3 (48.0)	50.9 (33.9)
Low-dose EPO	68.7 (71.8)*	32.4 (38.3)**	73.6 (65.9)
High-dose EPO	43.9 (36.8)**	30.2 (17.9)**	73.2 (53.5)
Transferrin saturation (%)§			
Placebo	30.9 (12.7)	27.2 (14.1)	28.7 (11.0)
Low-dose EPO	20.2 (8.1)**	12.6 (5.9)**	35.2 (13.8)
High-dose EPO	9.7 (3.2) **	12.1 (5.1)**	31.2 (17.9)

N = 16. Values are means (SD).

§ Data was log_10_ transformed before analysis.

*p<0.05;

**p<0.01 compared with placebo.

Both low-dose and high-dose Epoetin beta caused small, albeit statistically significant, increases in haematocrit, which with low-dose Epoetin beta extended onto day 25. Also Epoetin beta increased reticulocyte counts. The protocol did not include supplements of iron and as expected both doses of Epoetin beta decreased plasma levels of ferritin and transferrin saturation. All values, except the haematocrit value after low-dose Epoetin beta, had return to normal at day 25.

### Farmacokinetics

The elimination half-life of Epoetin beta is between 8 to 28 hours if subcutaneously administered [17], [18] and a maximal plasma concentration is obtained about 15 hours after injection [19]. The distribution volume corresponds to 1–2 times the plasma volume [18]. The terminal elimination rate constant β was calculated as ln2/t_1/2_. Assumed that the elimination rate constant β is a first order rate constant describing drug elimination from the body and Epoetin beta follows first order of kinetics in degradation, the expected contribution of Epoetin beta to the total plasma EPO concentration was estimated to be 386 mIU/ml, 155 mIU/ml, and 0.01 mIU/ml, respectively, with low-dose Epoetin beta at days 4, 11 and 25. Corresponding values with high-dose Epoetin beta were 3104 mIU/ml, 4 mIU/ml, and 0.00001 mIU/ml, respectively, at days 4, 11 and 25.

### Percentage of migrated isoforms (PMI)

**Figure 3:**
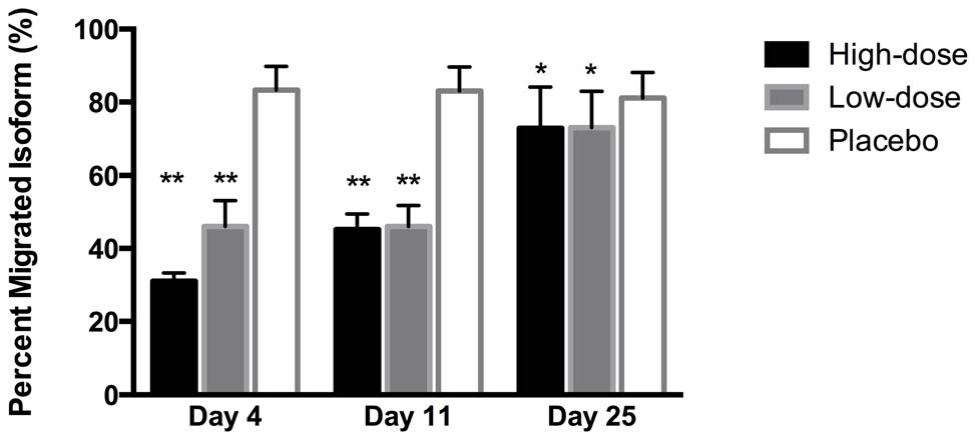
The percent migrated isoform at 15 mM GlcNAc. PMI after 4, 11 and 25 days of either high-dose rhEPO, low-dose rhEPO, or placebo. N = 15. Values are means with 95 % confidence intervals. *p<0.05; ** p<0.00001 compared with placebo.

In order to distinguish Epoetin beta from endogeneous EPO, the optimization curves demonstrates that the maximal deviation of PMI values was best obtained with 15 mM of GlcNAc as the W elution buffer *low* (Figure 2). With 15 mM of GlcNAc, PMI for EPO isoforms originating from normal plasma and umbilical cord plasma was 78.5 (3.4) % and 86.0 (9.4) %, respectively, whereas the PMI for Epoetin beta (300 ng/l) was 34.1 (7.8) (Figure 2). Both low-dose and high-dose administration of Epoetin beta caused a significant decrease in PMI values on all three days (Figure 3). PMI with placebo, reflecting the endogenous EPO isoforms, averaged 82.5 (10.3) % and remained unchanged at all days. High-dose Epoetin beta decreased PMI on days 4 and 11 to 31.0 (4.2) % (p<0.00001) and 45.2 (7.3) % (p<0.00001), respectively. PMI differed significantly between all days within the high-dose intervention (p<0.0001). Low-dose Epoetin beta decreased PMI on days 4 and 11 to 46.0 (12.8) % (p<0.00001) and 46.1 (10.4) % (p<0.00001), respectively. PMI after low-dose Epoetin beta did not differ between day 4 and 11, but were on both days significantly lower compared with day 25 (p<0.00001). In both rhEPO groups, PMI on day 25 was still decreased compared with placebo (Figure 3); high-dose Epoetin beta: 72.9 (19.4) (p<0.029) %; low-dose Epoetin beta: 73.1 (17.8) % (p<0.039).

**Figure 4:**
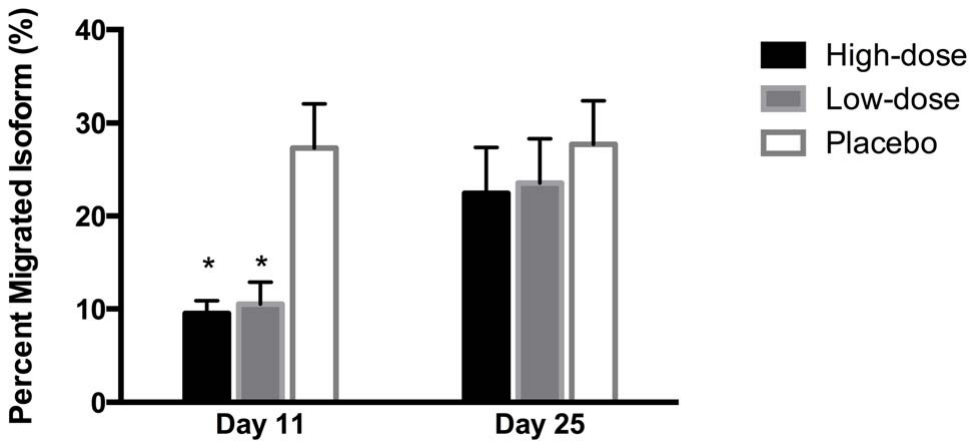
The percent migrated isoform at 5 mM GlcNAc. PMI after 11 and 25 days of either high-dose rhEPO, low-dose rhEPO, or placebo. N = 15. Values are means with 95 % confidence intervals. * p<0.00001 compared with placebo.

With 5 mM of GlcNAc, PMI of Epoetin beta (300 ng/l) was 4.8 (1.9) %, of normal plasma 17.2 (3.7) %, and of umbilical cord plasma 68.1 (16.0) % (Figure 2). PMI with placebo, reflecting the endogenous EPO isoforms, averaged 27.3 (8.5) % and 27.7 (8.4) % on day 11 and 25, respectively (Figure 4). On day 11, low-dose Epoetin beta and high-dose Epoetin beta decreased PMI to 10.5 (3.7) % (p<0.00001) and 9.5 (2.1) % (p<0.00001), respectively (Figure 4). On day 25, PMI did not differ between study days.

## Discussion

This prospective, double-blinded study demonstrates that the MAIIA test is able to detect a change in the distribution of EPO isoforms three weeks after the last injection of three daily high doses of Epoetin beta and 12 days after termination of a series of low dose Epoetin beta administrations. Noteworthy, the disruption of PMI was seen even though plasma EPO concentration and haematological parameters had normalized. The data suggest that plasma analysis by MAIIA may provide a sensitive measure of former intake of Epoetin beta.

The membrane assisted isoform immunoassay (MAIIA) assay was used in accordance with the principle outlined by Lönnberg *et al.* [11], [12], [20]. The MAIIA method was first described by Lönnberg *et al.* [11] as a rapid method for quantitative determination of low-abundance protein glycoisoforms in different types of biological samples. Compared with other methods, the sensitivity is high with a detection limit for EPO of 0.035 ng/l [21], [22]. Distinguishing between isoforms is based on the fact that differences in glycosylation between EPO isoforms in the presence of a low concentration of the competing GlcNAc solution results in fractionated release of isoforms from the WGA [7], [12], [22]. When expressed as percentage of migrated isoforms (PMI) in relation to the total amount of EPO that can be desorbed by a high GlcNAc solution, the method provides information about the relative distribution of circulating EPO isoforms. Because rhEPO isoforms bind more tightly to the WGA than endogenous isoforms, presence of rhEPO in a plasma sample results in a lower PMI than that measured in normal plasma. Even administration of very small doses of rhEPO has been reported to decrease PMI [23], [24]. In our subjects, PMI in normal plasma (placebo) averaged around 80 %, similar to values obtained in previous studies [12], [13].

By the use of samples from an earlier study [9], Lönnberg & Lundby [13] reported that a 28-days injection period of low-dose Epoetin beta significantly decreased PMI in plasma during the injection period. PMI had returned to normal values at 14 and 21 days after the last injection of Epoetin beta but was still significantly lower compared with baseline at day 7. The present data agree with this but indicate that the interval with significant alteration of PMI is at least 12 days. We added a series with short-term, high-dose rhEPO because this regime has been tested previously for its effects on central fatigue and cognition [25]–[27]. After three daily administration of high-dose Epoetin beta, PMI was still altered after 22 days, indicating a dose-dependent effect of Epoetin beta on circulating distribution of EPO isoforms. The discrepancy between studies may be due to the use of different concentrations of GlcNAc since the use of 10 mM of GlcNAc in the former study [13], as can be seen in Fig. 2, may result in lesser diversity between plasma with and without Epoetin beta. The optimization curves (Fig. 2) demonstrate that PMI, reflecting fractional specific desorptions of isoforms, depend upon the concentration of GlcNAc used in the low concentration. With 15 mM of GlcNAc, around 80 % of the EPO in normal plasma samples are desorbed, whereas PMI in soluted Epoitin beta was around 40 %, indicating that discrimination between endogenous EPO isoforms and Epoitin beta is best obtained with a GlcNAc 15 mM solution. Furthermore, the subjects in the present study served as their own control which eliminated inter-individual variation. Thus, our data suggest that optimization of MAIIA may favorably prolong the time span in which alteration of PMI can be used as an indirect marker of former use of Epoetin beta.

The main challenge in revealing doping with rhEPO is that the target effect, the increase in haemoglobin mass, is maintained for weeks after elimination of rhEPO [1], [2]. Even with the development of direct tests with higher sensitivity and specificity than available today, rhEPO in plasma and urine cannot be expected to be detectable for more than a few days after the last administration. As an alternative to direct testing, specific changes in haematological variables induced by former administration of rhEPO may emerge as changes in the ‘Athlete Biological Passport’ [4], [5], [28], [29]. Initial trials of the concept, however, reported that these indirect screening methods were able to correctly indicate rhEpo injections in only 58 % of the subjects [8]. Injections of very small doses of rhEPO sufficient to increase haemoglobin mass did not result in alteration of measures provided by the Athlete Biological Passport software [10]. In contrast, analysis with MAIIA after this injection regime of rhEPO demonstrated a reduction in PMI for up to 72 h after a microinjection [23], [24]. In the present study, plasma values of haemoglobin, ferritin, and tranferrin saturation and reticulocyte counts had all normalized at day 25, corresponding to 12 and 22 days after the last administration of low-dose and high-dose Epoetin beta, respectively. At the same time, PMI decreased in all subjects, suggesting that MAIIA provides a sensitive measure of the presence of an aberrant EPO isoform pattern at time points when circulating levels of Epoetin beta have fallen below the detection limit of direct tests.

MAIIA does not provide an exact molar measure of specific EPO isoforms but merely an indication of the relative binding to the lectin WGA in the actual pool of EPO isoforms. Presence of rhEPO isoforms in the sample will tend to lower the PMI, but the exact dose-response relationship remains unknown. Presumably, the present decrease in PMI is caused by remaining traces of Epoetin beta in plasma. In view of the farmacokinetics of Epoetin beta, however, it seems unlikely that there should be a significant contribution to the total EPO left at 22 and 12 days after last injection of high-dose Epoetin beta and low-dose Epoetin beta, respectively. The total EPO concentration on day 25 did not differ between series, also arguing against a contribution from earlier administred Epoetin beta. The N-glycans of both endogenous and recombinant EPO show a wide molecular variation so that the abundance of EPO consists of a mixture of isoforms [6], [30], [31]. Thus, another explanation for the suppression of PMI is that Epoetin beta causes a prolonged alteration in endogenous EPO synthesis and the isoform pattern. Previous studies have suggested that administration of rhEPO may trigger a negative feed-back mechanism that decreases the synthesis of renal EPO [14], [15], [32]. Epoetin beta causes a prompt decrease in plasma levels of renin and aldosterone, and renal clearance studies suggest that Epoetin beta decreases renal proximal tubular reabsorption rate leading to activation of the tubuloglomerular feedback mechanism and a fall in glomerular filtration rate [15]. Thus, treatment with rhEPO may result in suppression of endogenous EPO production through a decrease in intrarenal oxygen consumption [15], [32].

On the other hand, Epoetin beta also decreased PMI with the use of 5 mM of GlcNAc (Figure 4). At this concentration of GlcNAc, PMI in plasma from umbilical cords clearly deviates from values measured in normal plasma from adults (Figure 2) [16]. Whereas solutions of Epoitin beta consistently lowered PMI in relation to normal plasma, values in umbilical cord plasma were higher compared with normal plasma in adults. Most of the fetal EPO derives from the liver [33], [34] and EPO in umbilical cord plasma have be shown to differ from EPO in adults by the glycosylation pattern [35]. Thus, with 5 mM of GlcNAc, an increased hepatic production of EPO relative to the renal production should be expected to increase PMI [16]. The present finding that Epoetin beta decreased PMI at GlcNAc concentrations of both 5 and 15 mM argues against an effect of Epoetin beta on the relative contribution from endogenous sites of EPO synthesis. Taken together, the current study suggest that Epoetin beta causes an alteration in the EPO isoform pattern but the mechanisms remain unknown. Further studies are warranted to clarify the effect of rhEPO on endogenous EPO synthesis.

The administration time of low dose Epoetin beta was of shorter duration than is normally used to produce an increase in hemoglobin mass [36]. Furthermore, we did not include supplementation with dietary iron. Thus, the present results are not readily comparable with common practices in doping. Exposure to high altitude hypoxia did not change the EPO glycoform heterogeneity [16], but the effect, if any, of iron supplementation or autologous blood transfusion remains unknown. The present cross-over design, however, allowed a controlled evaluation of the effects of Epoetin beta *per se* on PMI obtained by MAIIA, and the effect of more prolonged administration of low dose Epoetin beta is likely to be enhanced. Still, it remains unknown whether other EPO related medical products can be traced by MAIIA and the results from this experimental and translational study cannot be extrapolated to an anti-doping setting. Although the detection sensitivity of MAIIA is superior to the currently accredited isoelectric focusing (IEF) for testing of athletes [12], [13], further large-scale studies are needed to establish the exact cut-off values and the true false positive and false negative rates.

In conclusion, this placebo-controlled, double-blinded, cross-over study suggest that administration of Epoetin beta in healthy subjects alters the EPO isoform distribution and leaves a footprint for up to three weeks after the last administration of rhEPO.

## Supporting Information

Data S1 **Raw data used in this study.**
(XLSX)
